# Association Between Allergen Sensitization and Anaphylaxis in Patients Visiting a Pediatric Emergency Department

**DOI:** 10.3389/fped.2021.651375

**Published:** 2021-06-08

**Authors:** Won Seok Lee, Lily Myung-Jin Cha, Man Yong Han, Kyung Suk Lee

**Affiliations:** ^1^Department of Pediatrics, CHA Ilsan Medical Center, CHA University, Goyang, South Korea; ^2^Department of Pediatrics, CHA Bundang Medical Center, CHA University, Seongnam, South Korea; ^3^Department of Pediatrics, Hanyang University Guri Hospital, Hanyang University, Gyeonggi-do, South Korea

**Keywords:** anaphylaxis, children, allergen, sensitization, emergency department

## Abstract

**Background and Objectives:** Anaphylaxis, a severe allergic disease, can be triggered by various causes. This study investigated the association between allergic sensitization and anaphylactic symptoms and the severity of anaphylaxis in children.

**Patients and Methods:** A retrospective review of 107 pediatric patients with anaphylaxis was performed between January 1, 2015, and December 31, 2017; 74 patients underwent allergen testing for specific immunoglobulin E. Allergic sensitizations and associations with anaphylactic symptoms and severity were investigated.

**Results:** Overall, 64 (86.5%) patients were sensitized to food or inhalant allergens. In children under 2 years of age, 90.5% were sensitized to food (*P* = 0.001); in those over 6 years of age, 84.6% were sensitized to inhalant allergens (*P* = 0.001). Milk sensitization was significantly associated with severe anaphylaxis (*P* = 0.036). The following symptoms showed significant associations with certain allergen sensitizations: facial edema with food; wheezing with milk; dyspnea with mite, etc. Certain allergen sensitizations presenting as risk factors for anaphylactic symptoms: wheat (adjusted odds ratio [aOR] = 4.644, *P* = 0.044) and nuts (aOR=3.614, *P* = 0.041) for wheezing, nuts (aOR=5.201, *P* = 0.026) for dyspnea, and milk (aOR=4.712, *P* = 0.048) for vomiting.

**Conclusion:** The allergen sensitization status differed according to the age of the children experiencing anaphylaxis. The severity, symptoms, and signs of anaphylaxis differed depending on the allergen sensitization status.

## Introduction

Anaphylaxis is an acute, life-threatening allergic disease characterized by a systemic hypersensitivity reaction in children (1). Emergency treatment is important for patients with anaphylaxis because severe symptoms can affect various organs within a short time (2). The lifetime prevalence of anaphylaxis is increasing (3) and the incidence of anaphylaxis in children is higher than that in adults (4).

Anaphylaxis presents with various symptoms with cutaneous symptoms (urticaria, angioedema) being the most common. Other symptoms include dyspnea, wheezing, syncope, and vomiting (3). The severity of anaphylaxis can be classified as mild, moderate, or severe according to the grading system issued by Brown (5). It is well-known that food is the most common trigger of anaphylaxis in children (6). Common food allergens are cow's milk in infants, peanuts in children, and tree nuts and shellfish in young adults (1, 6). A retrospective study revealed that food (85%), unknown causes (9%), drugs (6%), and insect stings (3%) were responsible for anaphylaxis at young ages (6), while in adult anaphylaxis, drugs and insect stings were found to be the most common agents with the most common class of drugs being the beta-lactam antibiotics, followed by non-steroidal anti-inflammatory drugs (7).

Many studies have analyzed the triggers of anaphylaxis, and some have indicated that anaphylactic symptoms could vary depending on the trigger of anaphylaxis (8, 9). A study of 382 cases of anaphylaxis in Poland found that drug/food-induced anaphylaxis caused more frequent skin and subcutaneous symptoms than respiratory symptoms and that Hymenoptera-induced anaphylaxis usually manifested as cardiovascular symptoms (81.4%) and cutaneous symptoms (76.6%) (8). In a study in Belgium, food was the most common trigger of anaphylaxis and about 40% of children had gastrointestinal symptoms compared to 21% in all age groups (9).

When the triggers of anaphylaxis are unclear, physicians might investigate the triggers using allergy tests, such as the Immuno CAP for allergen-specific IgE, the multiple allergen simultaneous test (MAST), and the allergic skin prick test (SPT). There has been no previous study on the allergen sensitization status in childhood anaphylaxis and its association with symptoms and severity. Therefore, this study aimed to investigate the association between allergen sensitization and anaphylactic symptoms, and the severity of anaphylaxis in children.

## Materials and Methods

### Patients

We analyzed the medical charts of 80,981 children and adolescents who visited a pediatric emergency center between January 1, 2015, and December 31, 2017. This pediatric emergency center focuses on urgent medical attention needed by children and adolescents under 15 years of age. Upon review, 146 children were confirmed as having anaphylaxis with International Classification of Disease (ICD) codes (T780, T782, T886).

Among the 146 patients, 107 children met the clinical criteria of Sampson et al. for the diagnosis of anaphylaxis (10). Of these, the 74 patients who underwent allergy tests were included in the study. This was a retrospective study and informed consent was not required. The study was approved by the Institutional Review Board of CHA University Bundang CHA Hospital (IRB number 2018-04-023). All the methods were carried out in accordance with relevant guidelines and regulations.

### Severity of Anaphylaxis and Specific IgE Evaluation

The severity of anaphylaxis was defined as mild, moderate, or severe according to the modified grading system developed by Brown (5). Mild anaphylaxis presents with cutaneous symptoms such as generalized edema, urticaria, periorbital edema, or angioedema. Moderate anaphylaxis shows features suggesting respiratory, cardiovascular, or gastrointestinal involvement (dyspnea, stridor, wheezing, throat or chest tightness, dizziness, diaphoresis, nausea, vomiting, abdominal pain, etc.). Severe anaphylaxis is associated with hypoxia (cyanosis or SpO2 ≤ 92% at any stage), hypotension (SBP < 90 mmHg in adults), or neurological compromises (confusion, collapse, loss of consciousness, incontinence, etc.) (5).

The following allergy evaluations were conducted in order to determine the patient's allergen sensitization status: ImmunoCAP (Thermo Fisher, Uppsala, Sweden), MAST (AdvanSure AlloScreen, Seoul, South Korea), and SPT. The majority of ImmunoCAP and MAST tests were conducted during the treatment of anaphylaxis in the emergency department. A few ImmunoCAP and MAST tests and all the SPTs were conducted at the outpatient clinic after management in the emergency department. The definition of a positive specific allergen followed the definition of previous studies (11, 12).

### Characteristics and Allergen Sensitizations

Seventy-four patients who underwent allergy evaluations were reviewed retrospectively. The following characteristics were reviewed: sex, age, history of allergic disease, family history of allergic disease, signs and symptoms, and specific allergen sensitization. Anaphylaxis, asthma, allergic rhinitis, atopic dermatitis, food allergies, drug allergies, and urticaria were reviewed to obtain the patient's history of allergic disease and to assess the patient's family allergy history. Urticarial rash, facial edema, throat tightness, dyspnea (a symptom complained of subjectively by the patient or caregiver), wheezing (a sign observed by a physician), abdominal pain, nausea and vomiting were analyzed as anaphylactic symptoms and signs. Egg, milk, nuts, wheat, and crustacean allergens were reviewed to assess food sensitization, and mite, animal, and tree allergens were reviewed to assess the inhalant sensitization status.

### Statistical Analysis

Continuous variables such as age were expressed in interquartile ranges because they were not normally distributed. Results were statistically analyzed using the Mann–Whitney *U*-test and the Fisher's exact test. A logistic regression analysis was conducted to identify the risk factors for anaphylactic symptoms. *P-*values of less than 0.05 were regarded as being statistically significant. All data were analyzed using SPSS version 25.0 software (IBM, Armonk, NY, USA).

## Results

### Characteristics and Comparison of Severity of Anaphylaxis

The clinical characteristics of all the patients are shown in Table 1. Among the patients with anaphylaxis who received an allergy evaluation, 47 (63.5%) were male and the median age was 4.0 years (interquartile range: 1.0–7.0). Fifty-eight (78.4%) patients had a history of allergic diseases and thirty-two (43.2%) had a family history of allergic diseases. The signs and symptoms were urticarial rash, present in 64 (86.5%) patients, dyspnea in 55 (74.3%), and facial edema in 50 (67.6%). There was no statistically significant difference in the severity of the clinical characteristics of the patients. The clinical characteristics of all patients among the age groups are shown in Supplementary Table 1.

**Table 1:** Clinical characteristics of the patients with anaphylaxis who underwent allergy tests (*n* = 74) and a comparison of the severity of anaphylaxis (mild to moderate vs. severe).

	**Total (%)**	**Mild to moderate (*n* = 56), (%)**	**Severe (*n* = 18), (%)**	*P*-value^*^
Sex (male) (%)	47 (63.5)	37 (66.1)	10 (55.6)	0.420
Age (median, years)	4.0 (1.0–7.0)	4.0 (1.0–8.0)	3.0 (1.0–5.0)	0.179
History of allergic disease (%)	58 (78.4)	43 (76.8)	15 (83.3)	0.557
Food allergy	33 (44.6)	24 (42.9)	9 (50.0)	0.786
Atopic dermatitis	24 (32.4)	18 (32.1)	6 (33.3)	1.000
Allergic rhinitis	23 (31.1)	17 (30.4)	6 (33.3)	1.000
Asthma	16 (21.6)	15 (26.8)	1 (5.6)	0.097
Anaphylaxis	8 (10.8)	6 (10.7)	2 (11.1)	1.000
Drug allergy	3 (4.1)	1 (1.8)	2 (11.1)	0.145
Urticaria	1 (1.4)	1 (1.8)	0 (0.0)	1.000
Family history of allergy (%)^†^	32 (43.2)	23 (41.1)	9 (50.0)	0.506
Anaphylaxis symptoms & signs				
Urticarial rash	64 (86.5)	49 (87.5)	15 (83.3)	0.607
Dyspnea	55 (74.3)	42 (75.0)	13 (72.2)	0.814
Facial edema	50 (67.6)	37 (66.1)	13 (72.2)	0.749
Wheezing	23 (31.1)	16 (28.6)	7 (38.9)	0.411
Vomiting	17 (23.0)	13 (23.2)	4 (22.2)	1.000
Throat tightness	10 (13.5)	7 (12.5)	3 (16.7)	0.167
Abdominal pain	6 (8.1)	6 (10.7)	0 (0.0)	0.629
Nausea	3 (4.1)	2 (3.6)	1 (5.6)	1.000

* *A comparison of the severity of anaphylaxis (mild to moderate vs. severe)*.

† *These include the anaphylaxis, asthma, allergic rhinitis, atopic dermatitis, food allergy, drug allergy, and urticarial history of the family*.

### Allergen Sensitization Rate and Comparison With the Severity of Anaphylaxis

**Figure 1:**
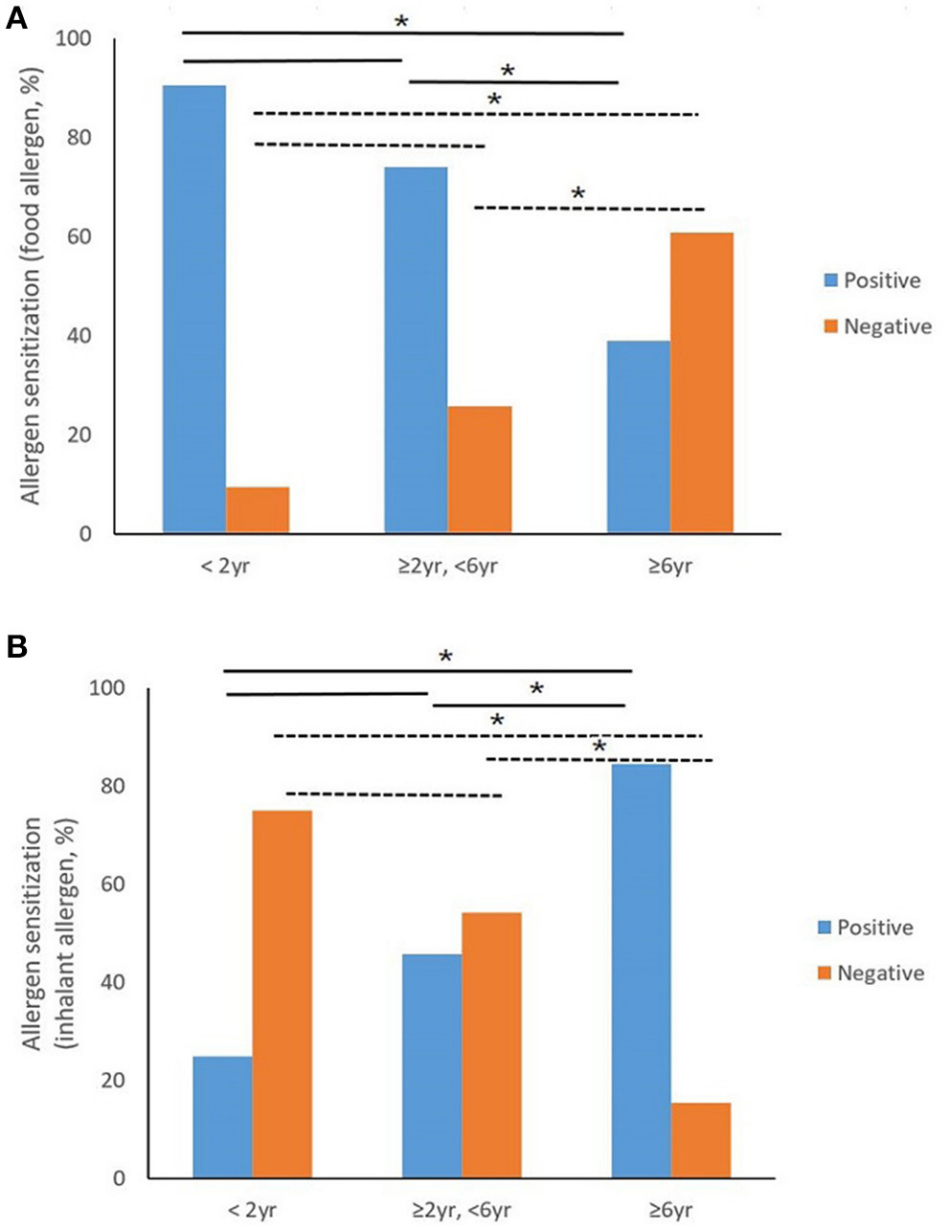
Trend of allergic sensitization by age group of children with anaphylaxis. **(A)** Shows the relationship between food allergen sensitization and age group. Food allergen sensitization rates were significantly different among the age groups (*P* = 0.001). With the Bonferroni correction, the food sensitization rate of the group aged over 6 years is significantly lower than that of other age groups. **(B)** Shows the relationship between inhalant allergen sensitization and age group. Inhalant allergen sensitization rates were significantly different among the age groups (*P* = 0.001). With the Bonferroni correction, the inhalant sensitization rate of the group over 6 years old was significantly higher than that of other age groups (*P < 0.05).

Sixty-four (86.5%) of a total of 74 patients were sensitized to food or inhalant allergens. The number of patients and allergen sensitization by the age group are shown in Supplementary Figure 1. Nineteen (90.5%) children under 2 years of age and nine (39.1%) children over 6 years of age were sensitized to food allergens with the food allergen sensitization rates being significantly different among the age groups (*P* = 0.001) (Figure 1A). Three (25.0%) children under 2 years of age and 22 (84.6%) children over 6 years of age were sensitized to inhalant allergens with the inhalant allergen sensitization rates being significantly different among the age groups (*P* = 0.001) (Figure 1B). Children under 2 years of age were more sensitized to food allergens than to inhaled allergens; the most sensitized allergens being egg followed by nuts, milk, and wheat, in that order. In the group of children aged ≥2 and <6 years, sensitization to nuts increased while sensitization to egg decreased. In the group aged ≥6 years, the rate of sensitization to inhalant allergens showed a significant increase, while on the other hand, the rate of sensitization to food allergens showed a significant decrease.

In our analysis of the sensitization rate of each allergen and the association between allergen sensitization and the severity of anaphylaxis (Table 2), 64 of the 74 patients (86.5%), were sensitized to at least one allergen. Forty-eight (67.6%) were sensitized to food allergens and 36 (58.1%) were sensitized to inhalant allergens. Eggs (50.8%) had the highest sensitization rate among foods, and mite (50.8%) had the highest rate among inhalant allergens. Severe anaphylaxis was associated significantly with milk sensitization (*P* = 0.036). Allergen sensitization and the severity of anaphylaxis among age groups are shown in Supplementary Table 2.

**Table 2:** Allergen sensitization rate of the allergy tests and the comparison with the severity of anaphylaxis (mild to moderate vs. severe) (*n* = 74).

	**Sensitization, (%)**	**Mild to moderate (*n* = 56), (%)**	**Severe (*n* = 18), (%)**	*P*-value^*^
All allergens	64/74 (86.5%)	50/56 (89.2%)	14/18 (77.8%)	0.214
All food	48/71 (67.6%)	35/54 (64.8%)	13/17 (76.5%)	0.370
Egg	32/63 (50.8%)	22/47 (46.8%)	10/16 (62.5%)	0.278
Milk	21/57 (36.8%)	13/44 (29.5%)	8/13 (61.5%)	0.036^†^
Nuts	29/60 (48.3%)	20/44 (45.5%)	9/16 (56.3%)	0.459
Wheat	14/49 (28.6%)	11/36 (30.6%)	3/13 (23.1%)	0.609
Crustacean	6/43 (10.3%)	3/34 (8.8%)	3/9 (33.3%)	0.059
All inhalants	36/62 (58.1%)	31/48 (64.6%)	5/14 (35.7%)	0.054
Mite	30/59 (50.8%)	26/46 (56.5%)	4/13 (30.8%)	0.101
Animal	12/47 (25.5%)	11/37 (29.7%)	1/10 (10.0%)	0.204
Tree	19/53 (35.8%)	15/40 (37.5%)	4/13 (30.8%)	0.660

* *A comparison of the severity of anaphylaxis (mild to moderate vs. severe)*.

† *P < 0.05*.

### Association Between Symptoms of Anaphylaxis and Type of Allergen Sensitization

An analysis of the association between anaphylactic symptoms and the type of allergen sensitization was conducted. Facial edema was associated with patients sensitized to food allergens (*P* = 0.038), and wheezing was associated with patients sensitized to milk (*P* = 0.025), wheat (*P* = 0.011), nuts (*P* = 0.034), and crustaceans (*P* = 0.036). Dyspnea was associated with patients sensitized to dust mites (*P* = 0.019) and tree pollen (*P* = 0.009) allergens, while vomiting was associated with food sensitization (*P* = 0.014) and milk allergens (*P* = 0.006) (Table 3). A comparison of anaphylactic triggers and allergen sensitizations, and comparison of anaphylactic triggers with the symptoms and sign of anaphylaxis are shown Supplementary Tables 3, 4.

**Table 3:** *P*-values of the Chi-square tests that analyzed the association between the symptoms of anaphylaxis and the type of allergen sensitization (*n* = 74).

	**Facial edema**	**Wheezing**	**Dyspnea**	**Vomiting**
	**(*n* = 47)**	**(*n* = 23)**	**(*n* = 55)**	**(*n* = 19)**
All allergens	0.340	0.416	0.736	0.188
All food	0.038^*^	0.086	0.628	0.014^*^
Egg	0.721	0.319	0.598	0.096
Milk	0.323	0.025^*^	0.5991	0.006^*^
Wheat	0.924	0.011^*^	0.609	0.335
Nuts	0.511	0.034^*^	0.204	0.118
Crustacean	0.402	0.036^*^	0.146	0.858
All inhalants	0.731	0.564	0.053	0.458
Mite	0.673	0.613	0.019^*^	0.205
Animal	0.562	0.961	0.083	0.240
Tree	0.279	0.302	0.009^*^	0.582

* *P < 0.05*.

The results of the logistic regression showed a relationship between the symptoms of anaphylaxis and the specific allergen sensitization as follows: wheezing was significantly associated with wheat (adjusted odds ratio [OR] = 4.644, *P* = 0.044) and nuts (adjusted OR=3.614, *P* = 0.041), dyspnea with nuts (adjusted OR = 5.201, *P* = 0.026), and vomiting with milk (adjusted OR = 4.712, *P* = 0.048) adjusted for sex, age, and familial allergy history (Table 4). However, there were no statistically significant associations between the severity of anaphylaxis and the type of allergen sensitizations from the results of the logistic regression analysis (data not shown).

**Table 4:** Logistic regression analysis of the association between the symptoms of anaphylaxis and the type of allergen sensitization (*n* = 74).

	**Wheezing**				**Dyspnea**				**Vomiting**			
	**cOR**	***P* value**	**aOR**	***P* value**	**cOR**	***P* value**	**aOR**	***P* value**	**cOR**	***P* value**	**aOR**	***P* value**
All allergen	1.953 (0.381–10.020)	0.422	2.247 (0.421–11.981)	0.343	1.286 (0.297–5.570)	0.737	1.658 (0.331–8.296)	0.538	3.522 (0.416–29.832)	0.248	3.825 (0.359–40.672)	0.266
All foods	2.850 (0.836–9.715)	0.094	3.192 (0.755–13.490)	0.115	0.748 (0.230–2.428)	0.629	3.425 (0.667–17.594)	0.140	13.200 (1.637–106.451)	0.015^*^	5.126 (0.536–49.011)	0.156
Egg	1.725 (0.588–5.063)	0.321	1.569 (0.363–6.781)	0.546	0.613 (0.189–1.992)	0.416	1.988 (0.405–9.756)	0.397	3.558 (1.083–11.683)	0.036^*^	0.916 (0.192–4.359)	0.912
Milk	3.766 (1.146–12.372)	0.029^*^	4.450 (0.998–19.841)	0.050	0.714 (0.209–2.445)	0.592	1.518 (0.350–6.576)	0.577	6.667 (1.947–22.830)	0.003^*^	4.712 (1.012–21.945)	0.048^*^
Wheat	5.333 (1.391–20.450)	0.015^*^	4.644 (1.046–20.626)	0.044^*^	1.467 (0.336–6.393)	0.610	2.471 (0.477–12.780)	0.281	1.875 (0.517–6.796)	0.339	1.173 (0.269–5.119)	0.831
Nuts	3.385 (1.069–10.724)	0.038^*^	3.614 (1.054–12.384)	0.041^*^	2.108 (0.660–6.734)	0.208	5.201 (1.219–22.185)	0.026^*^	2.420 (0.789–7.419)	0.122	1.684 (0.471–6.017)	0.423
Crustacean	6.222 (0.972–39.814)	0.054	5.029 (0.616–41.045)	0.132	-	-	-	-	1.182 (0.188–7.426)	0.859	0.733 (0.092–5.862)	0.769
All inhalant	0.726 (0.245–2.157)	0.565	0.991 (0.275–3.565)	0.988	3.125 (0.960–10.170)	0.058	1.340 (0.333–5.390)	0.680	0.543 (0.158–1.862)	0.331	4.588 (0.652–32.267)	0.126
Mite	1.347 (0.425–4.274)	0.613	2.349 (0.552–9.991)	0.248	4.062 (1.215–13.587)	0.023^*^	1.779 (0.422–7.502)	0.433	0.380 (0.111–1.298)	0.123	4.051 (0.503–32.596)	0.189
Animal	0.963 (0.213–4.362)	0.961	2.500 (0.366–17.069)	0.350	5.739 (0.660–49.906)	0.113	5.543 (0.513–59.909)	0.158	0.578 (0.106–3.153)	0.526	1.479 (0.157–13.900)	0.732
Tree	1.896 (0.558–6.444)	0.305	3.165 (0.697–14.371)	0.136	11.143 (1.325–93.686)	0.026	6.638 (0.706–62.398)	0.098	0.609 (0.141–2.638)	0.508	5.853 (0.525–65.248)	0.151

*cOR, crude odd ratio; aOR, adjusted odd ratio*.

* *P < 0.05, Adjusted by sex, age and familial history*.

## Discussion

This study evaluated specific allergen sensitizations in children with anaphylaxis and determined their associations with the symptoms of anaphylaxis. We found that the allergen sensitization status differed according to the age at which anaphylaxis was experienced in children. We observed that the severity, symptoms, and signs of anaphylaxis were different depending on allergen sensitization status. To our knowledge, this was the first study to evaluate the allergen sensitization status in children with anaphylaxis and its association with the severity and symptoms of anaphylaxis.

Anaphylaxis is a serious hypersensitivity allergic reaction, the molecular mechanisms of which are now well-known (1, 13). The activation of a signal cascade resulting in mast cell and basophil degranulation is important. These cells release multiple mediators including histamine, tryptase, and leukotrienes, resulting in various anaphylactic symptoms (14). Cytokines, such as the tumor necrosis factor-α, interleukin (IL)-4, IL-5, and IL-10, act as the contributing mediators in the development of various anaphylaxis symptoms (14).

In adults, drugs and insect stings are the most common triggers of anaphylaxis, and foods rank third (6). In contrast, the most common cause in children, is food (6). Recent studies revealed that emergency center visits and hospitalization due to food-induced anaphylaxis increased significantly in children (15). One study indicated that nuts, such as peanuts and cashews, were the most common food triggers in childhood anaphylaxis (6). Although these were not included in this study, the most common trigger in our patients was also food (54%) with the most common food triggers being nuts (25%), milk (25%), and egg (20%) (4). The results of our study confirmed that anaphylactic children were highly sensitized to these foods.

In our study, children under 2 years of age were more sensitized to food allergens than to inhalant allergens. In the group aged ≥2 and <6 years, sensitization to nuts increased while sensitization to egg decreased. In the group aged >6 years, the rate of sensitization to food allergens decreased dramatically, whereas the rate of sensitization to inhalant allergens increased significantly. This can be attributed to age-dependent differences and the natural course of allergy development and resolution (16). A study has reported that 19% of patients attained tolerance to milk by the age of 4 years, 42% by 8 years, and 79% by 16 years (17). With an egg allergy, 4% attained tolerance by the age of 4 years, 37% by 10 years, and 68% by 16 years (18). In contrast, allergies to peanuts, tree nuts, fish, and shellfish usually persist for a lifetime (19).

If allergen exposure in an anaphylactic event is apparent, the allergens can be presumed clinically to be triggering agents. However, it can be difficult to discern the causative agent, especially when simultaneous exposure is suspected. A meal usually contains various types of food and can also include food additives; of which any of the ingredients could be the causative trigger. An allergic evaluation would be required to identify the sensitized allergen in an anaphylactic event, especially when the trigger is uncertain. It may then be helpful to find the triggers with *in vivo* tests, such as skin and/or provocation tests, but these sometimes have potential risks for severe and even life-threatening allergic reactions, including anaphylaxis (20). Instead of direct provocations, *in vitro* tests, such as ImmunoCAP and MAST, can offer a complementary approach to identify the triggers of allergy (20–22). However, these tests have several limitations; inconsistencies and false-positive results are possible (12, 21, 22).

Although the most common trigger of childhood anaphylaxis is food, there was no difference in our study in the severity of anaphylaxis between those with or without a history of food allergy. In addition, histories of allergic diseases and family histories of allergic disease did not affect the severity of anaphylaxis in this study population. However, some previous studies have shown different results (23, 24). It has been reported that increased severity of asthma was associated with increased risk of anaphylaxis (23), and that patients with asthma had a higher risk of fatal food-induced anaphylaxis (24). More studies are needed to determine the associations between allergic diseases and the severity of anaphylaxis in children. We observed a significant association between severe anaphylaxis and milk sensitization. The sensitization status for inhalant allergens did not affect the severity of anaphylaxis in our patients.

Anaphylaxis can present with a variety of symptoms, and several studies have attempted to explain the associations between anaphylaxis triggers and symptoms (8, 25). In a study in Poland, the triggers of anaphylaxis were divided into foods, drugs, venoms, and latex, and the symptoms of each triggers were compared (8). Cutaneous symptoms were more common in food- and latex-triggered anaphylaxis, gastrointestinal symptoms in food-triggered cases, respiratory symptoms in venom-triggered cases and less common in latex-triggered cases, and cardiovascular symptoms were more common in venom-triggered cases and less common in food-triggered cases (8). In another study in France, most of the anaphylaxis that occurred in infancy was triggered by cow's milk (59.0%) (25). Cow's milk-induced anaphylaxis in infancy caused mucocutaneous symptoms such as hives, hypotonia, and hypotension, and those symptoms were more frequent in the infants than in older preschool children (25). While thus far, some studies have described the relationship between anaphylactic triggers and symptoms, to our knowledge, there have been no studies explaining the relationship between the allergic sensitization status determined by allergic tests and anaphylactic symptoms. From the findings of our study, there were significant associations between several allergen sensitization statuses and anaphylaxis symptoms such as facial edema, wheezing, dyspnea, and vomiting.

According to our results, in patients with allergic sensitization, we can consider the possibility of anaphylactic symptoms caused by specific triggers. Through efforts to educate patients and physicians, more rapid preparation and accurate treatment can be achieved for several urgent anaphylactic symptoms. Since anaphylaxis can present with various and sometimes ambiguous symptoms, the management of anaphylaxis requires prompt recognition and the early administration of an intramuscular epinephrine injection (1). In a study conducted in the USA, the food trigger was a risk factor for recurrent anaphylaxis-related emergency department visits; therefore, children who experienced food induced-anaphylaxis should carry self-injectable epinephrine (26). Therefore, parents and caregivers should be educated in the use of self-injectable epinephrine, with the periodic observation of symptoms, and an action plan for anaphylactic emergencies (27). In addition, a multidisciplinary training program aimed at preparedness for such emergencies should be offered to teachers and school caregivers (28). Parents and caregivers should also be informed of the predictive side effects of epinephrine (27). However, educating all parents how to use the self-injectable epinephrine may not be effective and safe. Instead, recognizing symptoms early and applying immediate and appropriate treatment by physicians appears more reasonable.

Our study had several limitations. It was difficult to prove a clear causal relationship because of the retrospective study design, and in addition, the number of patients was small. We could not conduct provocation tests to confirm the trigger allergens in order to prevent the occurrence of anaphylactic shock. Instead, we conducted specific allergic tests, which can be useful in the evaluation of a patient's sensitization.

In conclusion, specific allergen sensitization is related to the symptoms of anaphylaxis in children. Patients and physicians should be better instructed in emergency care, and in the prevention of anaphylactic events, according to the specific allergen sensitization in children with anaphylaxis. Further studies are required to analyze the mechanism between specific IgE and anaphylaxis in children and to understand the various allergen sensitization statuses and their associations with the symptoms and severity of anaphylaxis in childhood anaphylaxis. Using the results of the proposed future research, a model could be developed to predict anaphylaxis according to sensitization and, hence, enable the prevention of anaphylaxis in children and adolescents.

## Data Availability Statement

The original contributions generated for the study are included in the article/Supplementary Material, further inquiries can be directed to the corresponding author.

## Author Contributions

WL, MH, and KL conceived the idea. WL and KL analyzed the data and wrote the manuscript with input from all the authors. LC and MH investigated and supervised the findings of this study. All the authors discussed the results and contributed to the final manuscript.

## Conflict of Interest

The authors declare that the research was conducted in the absence of any commercial or financial relationships that could be construed as a potential conflict of interest.

## Supplementary Material

Supplementary Figure 1 The patient numbers of allergen sensitization by the age group. This figure showed the patients numbers of allergen sensitization by the age group.: 19 (90.5%) children under 2 years of age, 20 (74.1%) between 2 and 6 years of age, and 23 (39.1%) over 6 years of age were sensitized to food allergens, and three (25.0%) children under 2 years of age, 11 (45.8%) between 2 years and under 6 years old, and 22 (84.6%) over 6 years of age were sensitized to inhalant allergens. Children under 2 years of age were more sensitized to food allergens, and in the group aged ≥6 years, the rate of sensitization to inhalant allergens increased.

Supplementary Table 1 Clinical characteristics of patients with anaphylaxis who underwent allergy tests (*n* = 74) and a comparison of the severity of anaphylaxis (mild to moderate vs. severe).

Supplementary Table 2 Allergen sensitization rate of allergy tests and the severity of anaphylaxis (mild to moderate vs. severe) (*n* = 74).

Supplementary Table 3 Comparison of anaphylactic triggers with the allergic sensitizations of patients.

Supplementary Table 4 Comparison of anaphylactic triggers with the symptoms and sign of anaphylaxis.
