# Supplementary material for: Association Between Allergen Sensitization and Anaphylaxis in Patients Visiting a Pediatric Emergency Department
Source: Front Pediatr. 2021 Jun 8;9:651375. doi: 10.3389/fped.2021.651375 (PMC8217608; doi:10.3389/fped.2021.651375)
Supplement: Supplementary Figure 1 — The patient numbers of allergen sensitization by the age group. This figure showed the patients numbers of allergen sensitization by the age group.: 19 (90.5%) children under 2 years of age, 20 (74.1%) between 2 and 6 years of age, and 23 (39.1%) over 6 years of age were sensitized to food allergens, and three (25.0%) children under 2 years of age, 11 (45.8%) between 2 years and under 6 years old, and 22 (84.6%) over 6 years of age were sensitized to inhalant allergens. Children under 2 years of age were more sensitized to food allergens, and in the group aged ≥6 years, the rate of sensitization to inhalant allergens increased. [file Image_1.pdf]

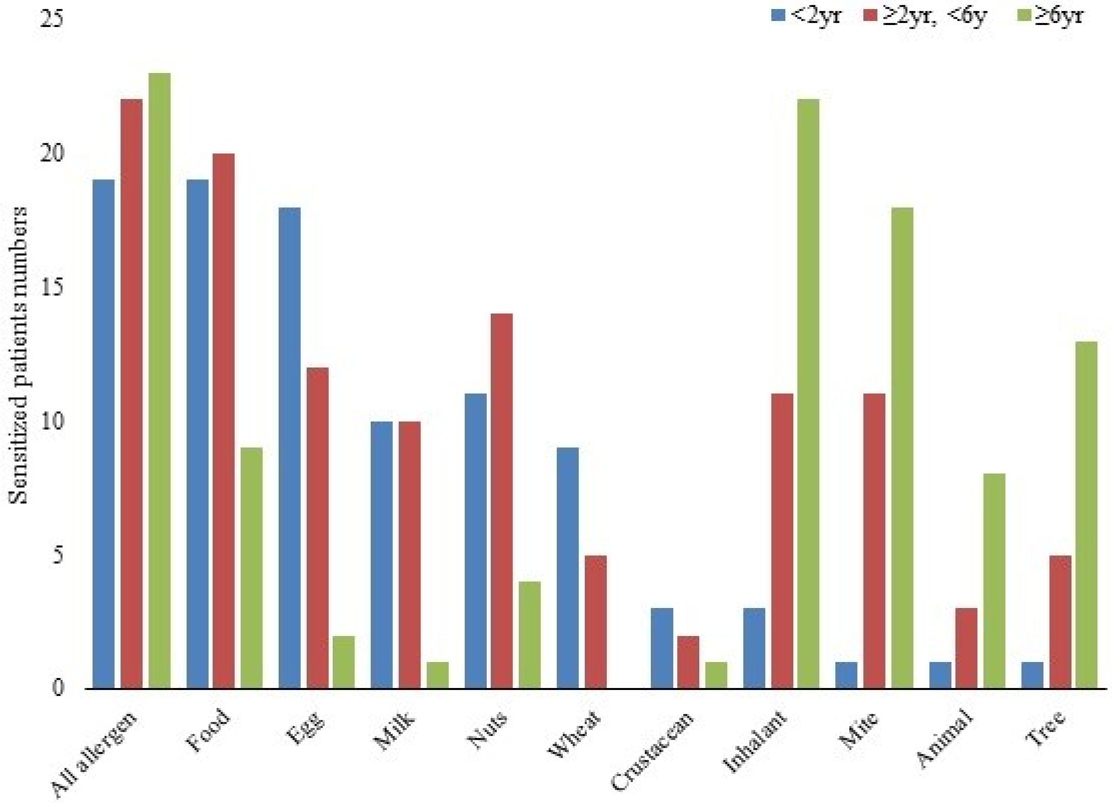

Supplementary Figure 1 | The patient numbers of allergen sensitization by the age group. This figure showed the patients numbers of allergen sensitization by the age group.: 19 (90.5%) children under 2 years of age, 20 (74.1%) between 2 and 6 years of age, and 23 (39.1%) over 6 years of age were sensitized to food allergens, and three (25.0%) children under 2 years of age, 11 (45.8%) between 2 years and under 6 years old, and 22 (84.6%) over 6 years of age were sensitized to inhalant allergens. Children under 2 years of age were more sensitized to food allergens, and in the group aged ≥6 years, the rate of sensitization to inhalant allergens increased.
