# Supplementary material for: Association Between Allergen Sensitization and Anaphylaxis in Patients Visiting a Pediatric Emergency Department
Source: Front Pediatr. 2021 Jun 8;9:651375. doi: 10.3389/fped.2021.651375 (PMC8217608; doi:10.3389/fped.2021.651375)
Supplement: Supplementary Table 1 — Clinical characteristics of patients with anaphylaxis who underwent allergy tests (n = 74) and a comparison of the severity of anaphylaxis (mild to moderate vs. severe). [file Data_Sheet_1.pdf]

Supplementary Table 1. Clinical characteristics of patients with anaphylaxis who underwent allergy tests (n=74) and a comparison of the severity of anaphylaxis (mild to moderate vs. severe)

| | Total, (%) (N=74) | Mild to moderate, (%) | | | | Severe, (%) | | | |
|---|---|---|---|---|---|---|---|---|---|
| | | <2yr=, (%) (n=15) | ≥2, <6yr, (%) (n=19) | ≥6yr, (%) (n=22) | Total, (%) (n=56) | <2yr, (%) (n=6) | ≥2, <6yr, (%) (n=8) | ≥6yr, (%) (n=4) | Total (%) (n=18) |
| Sex (male) (%) | 47 (63.5) | 12 (80.0) | 10 (52.6) | 15 (68.2) | 37 (66.1) | 4 (66.7) | 6 (75.0) | 0 (0.0) | 10 (55.6) |
| Age (median, years) | 4.0 (1.0-7.0) | 1.0 (0.0-1.0) | 4.0 (2.0-4.0) | 10.0 (6.0-13.0) | 4.0 (1.0-8.0) | 1.0 (0.0-1.0) | 3.0 (2.0-4.0) | 9.0 (7.0-9.0) | 3.0 (1.0-5.0) |
| History of allergic disease (%) | 58 (78.4) | 8 (53.3) | 16 (84.2) | 19 (86.4) | 43 (76.8) | 3 (50.0) | 8 (100.0) | 4 (100.0) | 15 (83.3) |
| Food allergy | 33 (44.6) | 7 (46.7) | 11 (57.9) | 6 (27.3) | 24 (42.9) | 2 (33.3) | 5 (62.5) | 2 (50.0) | 9 (50.0) |
| Atopic dermatitis | 24 (32.4) | 5 (33.3) | 7 (36.8) | 6 (27.3) | 18 (32.1) | 1 (16.7) | 4 (50.5) | 1 (25.0) | 6 (33.3) |
| Allergic rhinitis | 23 (31.1) | 0 (0.0) | 8 (42.1) | 9 (40.9) | 17 (30.4) | 0 (0.0) | 3 (37.5) | 3 (75.0) | 6 (33.3) |
| Asthma | 16 (21.6) | 0 (0.0) | 6 (31.6) | 9 (40.9) | 15 (26.8) | 0 (0.0) | 1 (12.5) | 0 (0.0) | 1 (5.6) |
| Anaphylaxis | 8 (10.8) | 0 (0.0) | 1 (5.3) | 5 (22.7) | 6 (10.7) | 0. (0.0) | 1 (12.5) | 1 (25.0) | 2 (11.1) |
| Drug allergy | 3 (4.1) | 0 (0.0) | 0 (0.0) | 1 (4.5) | 1 (1.8) | 0 (0.0) | 1 (12.5) | 1 (25.0) | 2 (11.1) |
| Urticaria | 1 (1.4) | 0 (0.0) | 0 (0.0) | 1 (4.5) | 1 (1.8) | 0 (0.0) | 0 (0.0) | 0 (0.0) | 0 (0.0) |
| Family history of allergy (%)† | 32 (43.2) | 7 (46.7) | 5 (31.6) | 11 (50.0) | 23 (41.1) | 3 (50.5) | 4 (50.0) | 2 (50.0) | 9 (50.0) |
| Anaphylaxis symptoms & signs | | | | | | | | | |
| Urticarial rash | 64 (86.5) | 14 (93.3) | 17 (89.5) | 18 (81.8) | 49 (87.5) | 6 (100.0) | 6 (75.0) | 3 (75.0) | 15 (83.3) |
| Dyspnea | 55 (74.3) | 9 (60.0) | 12 (63.2) | 21 (95.5) | 42 (75.0) | 4 (66.7) | 6 (75.0) | 3 (75.0) | 13 (72.2) |
| Facial edema | 50 (67.6) | 11 (73.3) | 13 (68.4) | 13 (59.1) | 37 (66.1) | 5 (83.3) | 5 (62.5) | 3 (75.0) | 13 (72.2) |
| Wheezing | 23 (31.1) | 4 (26.7) | 7 (36.8) | 5 (22.7) | 16 (28.6) | 2 (33.3) | 5 (62.5) | 0 (0.0) | 7 (38.9) |
| Vomiting | 17 (23.0) | 7 (46.7) | 6 (31.6) | 0 (0.0) | 13 (23.2) | 2 (33.3) | 2 (25.0) | 0 (0.0) | 4 (22.2) |
| Throat tightness | 10 (13.5) | 1 (6.7) | 2 (10.5) | 4 (18.2) | 7 (12.5) | 0 (0.0) | 1 (12.5) | 2 (50.0) | 3 (16.7) |
| Abdominal pain | 6 (8.1) | 0 (0.0) | 2 (10.5) | 2 (9.1) | 4 (7.1) | 0 (0.0) | 1 (12.5) | 1 (25.0) | 2 (11.0) |
| Nausea | 3 (4.1) | 0 (0.0) | 1 (5.3) | 1 (4.5) | 2 (3.6) | 0 (0.0) | 0 (0.0) | 1 (25.0) | 1 (5.6) |

* A comparison of the severity of anaphylaxis (mild to moderate vs. severe).
† these include the anaphylaxis, asthma, allergic rhinitis, atopic dermatitis, food allergy, drug allergy, and urticarial history of the family.

Supplementary Table 2. Allergen sensitization rate of allergy tests and the severity of anaphylaxis (mild to moderate vs. severe) (n=74)

| | Total (n=74), (%) | Mild to moderate (n=56), (%) | | | | Severe (=18), (%) | | | |
|---|---|---|---|---|---|---|---|---|---|
| | | <2yr, (%) | ≥2, <6yr, (%) | ≥6yr, (%) | Total, (%) | <2yr, (%) | ≥2, <6yr, (%) | ≥6yr, (%) | Total, (%) |
| All allergens | 64/74 (86.5%) | 15/15 (100.0) | 16/19 (84.2) | 19/22 (86.4) | 50/56 (89.2%) | 4/6 (66.7) | 6/8 (75.0) | 4/4 (100.0) | 14/18 (77.8%) |
| All food | 48/71 (67.6%) | 15/15 (100.0) | 14/19 (73.7) | 6/20 (30.0) | 35/54 (64.8%) | 4/6 (66.7) | 6/8 (75.0) | 3/3 (100.0) | 13/17 (76.5%) |
| Egg | 32/63 (50.8%) | 14/15 (93.3) | 7/17 (41.1) | 1/15 (6.7) | 22/47 (46.8%) | 4/6 (66.7) | 5/7 (71.4) | 1/3 (33.3) | 10/16 (62.5%) |
| Milk | 21/57 (36.8%) | 7/15 (46.7) | 6/14 (42.9) | 0/15 (0.0) | 13/44 (29.5%) | 3/6 (50.0) | 4/8 (50.0) | 1/4 (25.0) | 8/13 (61.5%) |
| Nuts | 29/60 (48.3%) | 9/13 (69.2) | 8/16 (50.0) | 3/15 (20.0) | 20/44 (45.5%) | 2/5 (40.0) | 6/8 (75.0) | 1/3 (33.3) | 9/16 (56.3%) |
| Wheat | 14/49 (28.6%) | 2/11 (22.2) | 4/12 (33.3) | 5/13 (38.5) | 11/36 (30.6%) | 0/4 (0.0) | 1/4 (25.0) | 2/5 (40.0) | 3/13 (23.1%) |
| Crustacean | 6/43 (10.3%) | 3/10 (30.0) | 0/11 (0.0) | 0/13 (0.0) | 3/34 (8.8%) | 0/2 (0.0) | 2/3 (66.7) | 1/4 (25.0) | 3/9 (33.3%) |
| All inhalants | 36/62 (58.1%) | 3/8 (37.5) | 10/18 (55.6) | 18/22 (81.8) | 31/48 (64.6%) | 0/4 (0.0) | 1/6 (16.7) | 4/4 (100.0) | 5/14 (35.7%) |
| Mite | 30/59 (50.8%) | 1/10 (10.0) | 10/17 (58.8) | 15/19 (78.9) | 26/46 (56.5%) | 0/3 (0.0) | 1/6 (16.7) | 3/4 (75.0) | 4/13 (30.8%) |
| Animal | 12/47 (25.5%) | 1/5 (20.0) | 3/13 (23.1) | 7/19 (36.8) | 11/37 (29.7%) | 0/3 (0.0) | 0/3 (0.0) | 1/4 (25.0) | 1/10 (10.0%) |
| Tree | 19/53 (35.8%) | 1/5 (20.0) | 4/15 (26.7) | 10/20 (50.0) | 15/40 (37.5%) | 0/4 (0.0) | 1/5 (20.0) | 3/4 (75.0) | 4/13 (30.8%) |

*A comparison of the severity of anaphylaxis (mild to moderate vs. severe).

†$P < 0.05$

Supplementary Table 3. Comparison of anaphylactic triggers with the allergic sensitizations of patients

| | All allergen sensitization | All food sensitization | Egg sensitization | Milk sensitization | Nuts sensitization | Wheat sensitization | Crustacean sensitization | All inhalant sensitization | Mite sensitization | Animal sensitization | Tree sensitization |
|---|---|---|---|---|---|---|---|---|---|---|---|
| **Triggers** | | | | | | | | | | | |
| Food (n=41) | 39 (95.1%) | 35 (85.4%) | 24 (58.5%) | 16 (39.0%) | 21 (51.2%) | 9 (22.0%) | 3 (7.3%) | 16 (39.0%) | 13 (31.7%) | 35 (85.4%) | 24 (58.5%) |
| Immune therapy (n=8) | 8 (100.0%) | 3 (37.5%) | 2 (25.0%) | 0 (0.0%) | 1 (12.5%) | 0 (0.0%) | 1 (12.5%) | 8 (100.0%) | 8 (100.0%) | 5 (62.5%) | 5 (62.5%) |
| Exercise (n=3) | 3 (100.0%) | 1 (33.3%) | 0 (0.0%) | 0 (0.0%) | 1 (33.3%) | 0 (0.0%) | 0 (0.0%) | 3 (100.0%) | 1 (33.3%) | 1 (33.3%) | 3 (100.0%) |
| Drug (n=4) | 2 (50.0%) | 0 (0.0%) | 0 (0.0%) | 0 (0.0%) | 0 (0.0%) | 0 (0.0%) | 0 (0.0%) | 2 (50.0%) | 1 (25.0%) | 1 (25.0%) | 1 (25.0%) |
| **Food triggers** | | | | | | | | | | | |
| Egg (n=6) | 6 (100.0%) | 6 (100.0%) | 6 (100.0%) | 1 (16.7%) | 0 (0.0%) | 2 (33.3%) | 0 (0.0%) | 1 (16.7%) | 0 (0.0%) | 1 (16.7%) | 0 (0.0%) |
| Milk (n=8) | 7 (87.5%) | 7 (87.5%) | 7 (87.5%) | 6 (75.0%) | 5 (62.5%) | 1 (12.5%) | 0 (0.0%) | 0 (0.0%) | 0 (0.0%) | 0 (0.0%) | 0 (0.0%) |
| Nuts (n=11) | 10 (90.9%) | 9 (81.8%) | 3 (27.3%) | 2 (18.2%) | 8 (72.7%) | 0 (0.0%) | 1 (9.1%) | 6 (54.5%) | 6 (54.5%) | 1 (9.1%) | 1 (9.1%) |
| Wheat (n=3) | 3 (100.0%) | 3 (100.0%) | 1 (33.3%) | 1 (33.3%) | 2 (66.7%) | 2 (66.7%) | 1 (33.3%) | 0 (0.0%) | 0 (0.0%) | 0 (0.0%) | 0 (0.0%) |
| Crustacean (n=2) | 2 (100.0%) | 2 (100.0%) | 1 (50.0%) | 1 (50.0%) | 1 (50.0%) | 0 (0.0%) | 1 (50.0%) | 0 (0.0%) | 0 (0.0%) | 0 (0.0%) | 0 (0.0%) |
| Buckwheat (n=1) | 1 (100.0%) | 1 (100.0%) | 0 (0.0%) | 0 (0.0%) | 0 (0.0%) | 0 (0.0%) | 0 (0.0%) | 1 (100.0%) | 0 (0.0%) | 0 (0.0%) | 1 (100.0%) |
| Fish (n=1) | 1 (100.0%) | 0 (0.0%) | 0 (0.0%) | 0 (0.0%) | 0 (0.0%) | 0 (0.0%) | 0 (0.0%) | 1 (100.0%) | 1 (100.0%) | 0 (0.0%) | 0 (0.0%) |
| Unspecified foods (n=9) | 6 (100.0%) | 6 (100.0%) | 6 (100.0%) | 1 (16.7%) | 5 (55.6%) | 4 (44.4%) | 0 (0.0%) | 1 (16.7%) | 0 (0.0%) | 1 (16.7%) | 0 (0.0%) |

Supplementary Table 4. Comparison of anaphylactic triggers with the symptoms and sign of anaphylaxis

| | Urticarial rash | Facial edema | Throat tightness | Dyspnea | Wheezing | Hypotension | Abdominal pain | Vomiting |
|---|---|---|---|---|---|---|---|---|
| **Triggers** | | | | | | | | |
| Food (n=41) | 35 (85.4%) | 29 (70.7%) | 7 (17.1%) | 28 (68.3%) | 17 (41.5%) | 4 (9.8%) | 4 (9.8%) | 17 (41.5%) |
| Immune therapy (n=8) | 6 (75.0%) | 3 (37.5%) | 0 (0.0%) | 8 (100.0%) | 3 (37.5%) | 0 (0.0%) | 0 (0.0%) | 0 (0.0%) |
| Exercise (n=3) | 3 (100.0%) | 2 (66.7%) | 1 (33.3%) | 3 (100.0%) | 1 (33.3%) | 0 (0.0%) | 0 (0.0%) | 0 (0.0%) |
| Drug (n=4) | 1 (25.0%) | 3 (75.0%) | 1 (25.0%) | 2 (50.0%) | 0 (0.0%) | 1 (25.0%) | 0 (0.0%) | 0 (0.0%) |
| **Food triggers** | | | | | | | | |
| Egg (n=6) | 5 (83.5%) | 4 (66.7%) | 0 (0.0%) | 3 (50.0%) | 1 (16.7%) | 0 (0.0%) | 0 (0.0%) | 2 (33.3%) |
| Milk (n=8) | 6 (75.0%) | 4 (50.0%) | 1 (12.5%) | 4 (50.0%) | 3 (37.5%) | 1 (12.5%) | 0 (0.0%) | 6 (75.0%) |
| Nuts (n=11) | 11 (100.0%) | 9 (81.8%) | 3 (27.3%) | 5 (45.5%) | 4 (36.4%) | 3 (27.3%) | 1 (9.1%) | 5 (45.5%) |
| Wheat (n=3) | 3 (100.0%) | 3 (100.0%) | 0 (0.0%) | 3 (100.0%) | 3 (100.0%) | 0 (0.0%) | 0 (0.0%) | 1 (33.3%) |
| Crustacean (n=2) | 2 (100.0%) | 2 (100.0%) | 0 (0.0%) | 2 (100.0%) | 2 (100.0%) | 0 (0.0%) | 1 (50.0%) | 0 (0.0%) |
| Buckwheat (n=1) | 1 (100.0%) | 1 (100.0%) | 1 (100.0%) | 1 (100.0%) | 0 (0.0%) | 0 (0.0%) | 0 (0.0%) | 0 (0.0%) |
| Fish (n=1) | 1 (100.0%) | 1 (100.0%) | 1 (100.0%) | 1 (100.0%) | 0 (0.0%) | 0 (0.0%) | 0 (0.0%) | 0 (0.0%) |
| Unspecified foods (n=9) | 6 (66.7%) | 5 (55.6%) | 1 (11.1%) | 9 (100.0%) | 4 (44.4%) | 0 (0.0%) | 2 (22.2%) | 3 (33.3%) |
